# A Retrospective 5-Year Single Center Study Highlighting the Risk of Cancer Predisposition in Adolescents and Young Adults

**DOI:** 10.3390/cancers13123033

**Published:** 2021-06-17

**Authors:** Frank Jordan, Simon Huber, Sebastian Sommer, Gerhard Schenkirsch, Michael C. Frühwald, Martin Trepel, Rainer Claus, Michaela Kuhlen

**Affiliations:** 1 Department of Hematology and Clinical Oncology, University Medical Center Augsburg, 86156 Augsburg, Germany; frank.jordan@uk-augsburg.de (F.J.); sebastian.sommer@uk-augsburg.de (S.S.); martin.trepel@uk-augsburg.de (M.T.); rainer.claus@uk-augsburg.de (R.C.); 2 Paediatrics and Adolescent Medicine, University Medical Center Augsburg, 86156 Augsburg, Germany; simon.huber@uk-augsburg.de (S.H.); michael.fruehwald@uk-augsburg.de (M.C.F.); 3 Comprehensive Cancer Center Augsburg, University Medical Center Augsburg, 86156 Augsburg, Germany; gerhard.schenkirsch@uk-augsburg.de

**Keywords:** adolescents, young adults, inherited cancer predisposition, awareness, suspicious findings

## Abstract

**Simple Summary:**

Genetic disposition to malignancies represents a significant factor in the diagnosis and treatment of cancer patients, as well as for pre- and post-treatment care of patients and their relatives. In our work, we point out the specific distribution of malignancies in adolescents and young adult patients (AYAs), highlighting the association with an increased risk of genetic predisposition. Genetic disposition requires special attention, especially for AYA patients. Knowledge of an underlying inherited cancer susceptibility facilitates individualized therapies and targeted efforts in cancer surveillance and prevention. With our work, we seek to contribute to a more consistent integration of the screening of AYAs for hereditary cancer predisposition into daily practice in the future.

**Abstract:**

The knowledge of inherited cancer susceptibility opens a new field of cancer medicine. We conducted a retrospective single-center cohort study. Data of AYA cancer patients registered between January 2014 and December 2018 were analyzed. The median age at cancer diagnosis of 704 patients (343 males, 361 females) was 32 years (range, 15–39 years), median follow-up was 181 days (range, 1–1975 days). Solid tumors were diagnosed in 575 (81.7%) patients, hematologic malignancies in 129 (18.3%) patients. Multiple primary cancers were reported in 36 (5.1%) patients. Malignancies that may be indicators of inherited cancer susceptibility were diagnosed in 2.6% of patients with cancers of the endocrine system, in 73% of cancers of the gastrointestinal system, in 88% of tumors of the central nervous system, in 92% of cancers of the urinary tract, and in 59% of head and neck tumors. In addition, all patients with breast cancer, sarcoma, and peripheral nerve sheath tumor were in need of genetic counselling. In sum, at least 181 of 704 (25.7%) AYA cancer patients presented with malignancies suspicious of harboring pathogenic germline variants. Evaluation of AYA cancer patients for hereditary cancer predisposition needs to be integrated into daily practice.

## 1. Introduction

Cancer genetics is increasingly integrated into practice in oncology [1]. This greatly advanced our understanding of hereditary cancers and the number of identified patients at risk. The hereditary burden of cancer due to pathogenic germline variants in adults ranges from 3% to 12.6% [2,3,4,5].

The selection for genetic testing of an underlying cancer predisposition syndrome (CPS) is traditionally based on pathologic features of the tumor, age at diagnosis, and family history [6,7]. The proportion of patients fulfilling criteria of clinical guidelines for genetic testing (e.g., multiple primary malignancies) who actually receive genetic testing in clinical practice, however, is poorly studied. In addition, these indicators may be non-indicative in a proportion of affected patients [8]. Indeed, recent tumor-normal sequencing studies demonstrated increased detection of individuals with potentially clinically significant heritable mutations over the predicted yield of targeted germline testing based on current clinical guidelines [9,10,11]. In addition, a number of tumor types highly suggestive of an underlying CPS were recognized which are not yet integrated in clinical guidelines [9,10,12].

The knowledge of inherited cancer susceptibility has opened a new field of cancer medicine including cancer risk assessment, individualized therapies, and targeted efforts in cancer surveillance and prevention [13,14]. In addition, it facilitates genetic testing in at-risk relatives and genetic counselling regarding family planning [15,16].

Adolescents and young adults (AYAs) (aged 15–39 years) represent a unique patient cohort that merits special considerations including underlying etiological factors and tumor biology [17,18,19].

AYA cancers are very heterogenous and may include ‘pediatric’ cancer occurring at older than expected ages as well as adult-onset cancer occurring at unusually young ages (an indicator of a hereditary CPS) [17,19]. Improved probability of survival and cure necessitates focus on long-term complications including secondary malignant neoplasm (SMN) and the impact of cancer on quality of life, fertility and family planning considerations [18,20].

Cancer incidence in AYAs is increasing over time [21,22,23]. About 17,000 individuals per year are diagnosed in Germany and approximately 70,000 in the USA. The most common types of cancer in AYAs include Hodgkin´s disease, melanoma, cancers of the breast, endocrine and genital system including germ cell tumors [12,24,25]. The prevalence of cancer predisposing germline variants (PGVs) in early-onset (adult) cancers amounts up to 23% in breast cancer [26], 35% in colorectal cancer [27], and 13% in certain sarcomas [28].

Early recognition of underlying CPS in AYAs may facilitate individualized therapies (e.g., avoiding irradiation and genotoxic chemotherapy in patients with Li-Fraumeni syndrome [29], preference for platinum-based therapy [30] and PARP inhibition in patients with homologous recombination repair deficiency [31]) and adaptation of tumor surveillance for early detection of subsequent tumors [32,33]. It thereby offers the potential to reduce morbidity and mortality. In addition, genetic diagnosis enables genetic testing of at-risk family members prior to the development of a first malignancy [15,32]. It further opens a number of options in carriers of pathogenic variants for family planning, which is particularly relevant to this age group [34].

Last year, data on the prevalence of germline susceptibility on AYA patients were presented at the American Association for Cancer Research Virtual Annual Meeting II [12]. Germline genetic testing was performed in 1201 patients with various solid tumors refined as early-onset (*n* = 877; 73%) and young-adult (*n* = 324; 27%) cancer cases (for definitions, please refer to the methods section). In the early-onset cancer cohort, the prevalence of pathogenic or likely pathogenic (P/LP) variants was 21% vs. 13% in the young adult cancer cohort. The most commonly mutated genes were *BRCA2*, *BRCA1*, *CHEK2*, and *ATM*. Pancreatic, breast, and kidney cancers harbored the highest rates of PGVs. In contrast, in the young adult cancer cohort, *TP53* and *SDHA* were most commonly mutated. Noteworthy, among young adults with sarcoma, the mutation prevalence was 18.1% and, thus, similar to the early-onset group.

To elucidate cancer diagnosis in and to identify at-risk AYA cancer patients (further referred to as AYAs), we conducted a retrospective single-center cohort study in a tertiary-care hospital. The overall aim of the study was (1) to determine the number of and cancer types in AYAs, (2) to compare cancer diagnosis in these AYAs with published data on the prevalence of cancer predisposing germline variants in that cancer type, and (3) to identify at-risk AYAs to provide information and recommend genetic counseling. Finally, we endeavor to raise awareness and to assist clinicians to recognize AYAs who require evaluation for an underlying CPS.

## 2. Materials and Methods

Data of patients aged 15 to 39 years, who were registered in the Augsburg Tumor Data Management (ATDM) of the Comprehensive Cancer Center Augsburg (CCCA) between 1 January 2014 and 31 December 2018 were analyzed. Data cut-off and end of follow-up was 21 January 2020. The average length of time from diagnosis to capture in the ATDM registry was 129.3 days (range, 0–1751). The ATDM of the CCCA includes all cancer cases of the catchment area of Swabia (population approximately 1.8 million) referred to and/or treated at the CCCA.

Patients´ diagnoses were documented in the ATDM based on ICD-10-codes. This analysis was restricted to patients with malignant neoplasms coded in ICD-10 group C. Patients with diagnosis in ICD-10 group D that is in-situ neoplasm, benign neoplasm, and neoplasm of uncertain behavior were excluded from this analysis. Data collection comprised demographic and clinical information including sex, age, diagnosis of the malignancy and data of last follow-up. Family history was assessed by the treating physician usually according to clinical routine assessment procedures usually based on tumor entity specific guidelines for familial cancer and genetic testing, respectively (e.g., German Consortium for Hereditary Breast and Ovarian Cancer).

According to the regional law (Bayerisches Krankenhausgesetz (BayKrG) in the version of 28 March 2007 (GVBl. S. 288, BayRS 2126-8-G Art. 27 Abs4), informed consent for retrospective analysis of the data is not necessary.

To make our data comparable to data of the Centre for Cancer Registry (GCR) of the Robert-Koch-Institute (RKI), Germany, records were organized by ICD10-based diagnosis groups analog to the Cancer Registry for AYA patients (age 15 to 39 years) from 1999 to 2016 [35]. According to this, neuroendocrine tumors were classified considering its origin.

Analogous to the definition used by Stadler et al. [12], yet provided as abstract only, we defined early onset cancer (EO-CA) as cancer wherein age 39 years is more than one standard deviation below the mean age of diagnosis for that cancer type. Young-adult cancer (YA-CA) was defined as cancer wherein age 39 years is less than one standard deviation below the mean age at cancer diagnosis. Mean age and standard deviation for each cancer type were extracted from the SEER data [24].

From recent literature, we extracted a data overview on the prevalence of germline mutations in patients, particularly young adults, with cancer. (Table S1) Based on this, we compiled a list of types of cancers (Table 1), which had been associated with a higher CPS rate. For this analysis, we manually reviewed ICD-based diagnoses and free text entries of AYA cancer patients registered in the ATDM and identified those patients potentially needing referral to genetic counselling due to their specific type of cancer.

*p* values for differences of prevalences between patient subgroups in distinct tumor subtypes were assessed by Chi-squared testing or Fisher’s exact test for small sample sizes, respectively.

## 3. Results

**Figure 1:**
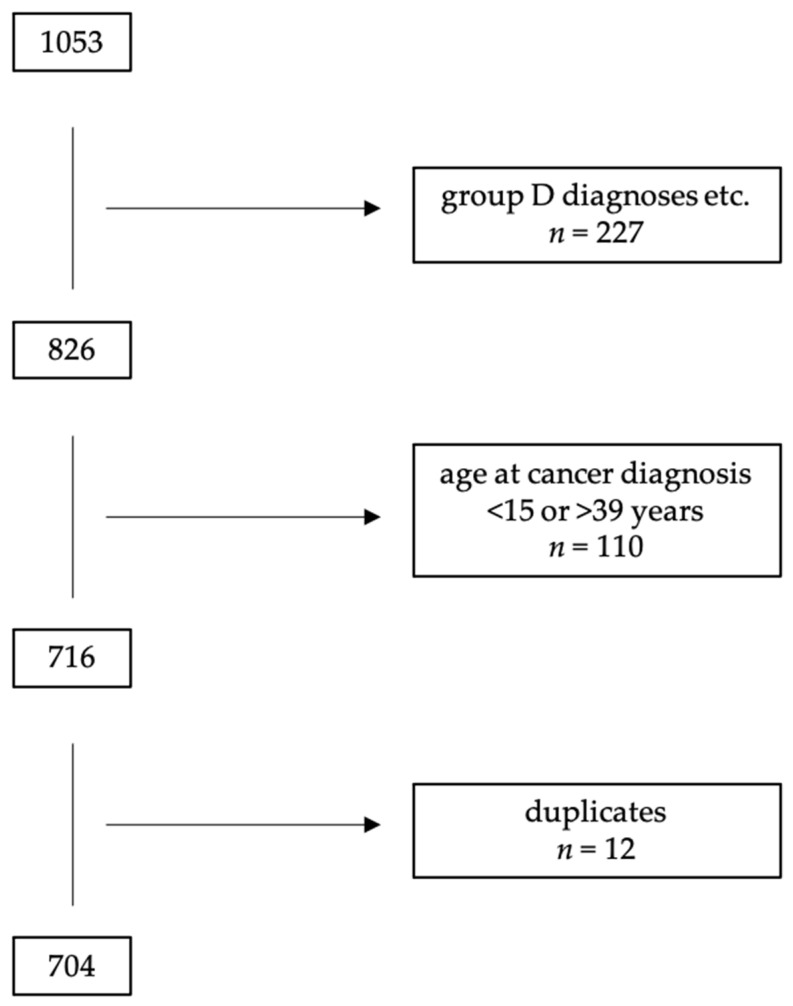
Consort diagram. Group D diagnoses: in-situ neoplasm, benign neoplasm, neoplasm of uncertain behavior.

A total of 1053 AYAs were registered in the ATDM database; 349 patients were ultimately excluded, leaving a final 704 (66.9%) patients eligible for this analysis (shown in Figure 1).

The median age at cancer diagnosis was 32 years (range, 15–39 years), median follow-up was 181 days (range, 1–1975 days). Sex ratio was balanced in the study cohort (343 (48.7%) males and 361 (51.3%) females). Of 704 patients, 19 females and 26 males (*n* = 45, 6.4%) died within the study period.

**Figure 2:**
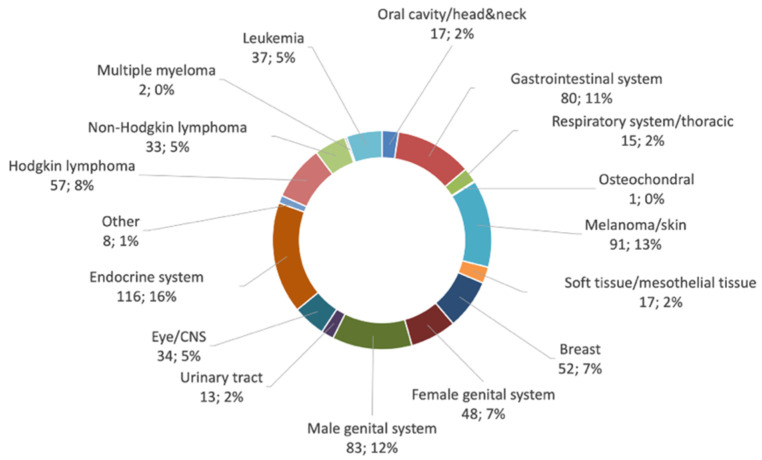
Diagnostic groups of solid and hematologic malignancies and their relative proportion of all invasive cancers that occurred in 704 15- to 39-year-old patients registered by the Augsburg Tumor Data Management of the Comprehensive Cancer Center Augsburg from 2014 to 2018 are shown in the ring diagram.

Solid tumors were diagnosed in 575 (81.7%) patients, 129 (18.3%) patients presented with hematologic malignancies (shown in Figure 2). Endocrine tumors accounted for the largest proportion (*n* = 116, 16.5%) of all cases, with thyroid carcinoma alone accounting for 16.2% (*n* = 114) of all cases. Second most frequent neoplasia was melanoma (*n* = 91, 12.9%) followed by lymphoma (*n* = 90, 12.8%) including 63.3% (*n* = 57) Hodgkin lymphoma cases.

**Figure 3:**
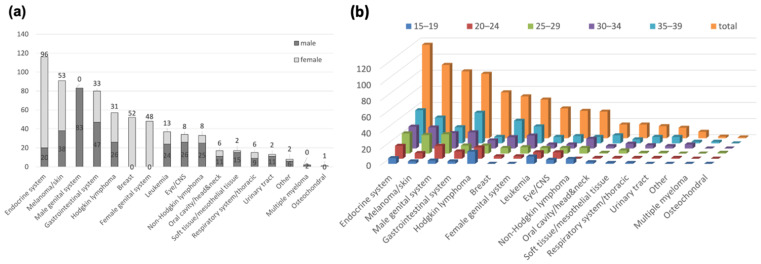
The distribution of the most frequent cancers in the Augsburg Tumor Data Management of the Comprehensive Cancer Center Augsburg from 2014 to 2018 among 15- to 39-year olds according to gender (**a**) and by 5-year age intervals (**b**) are shown in the bar charts.

The distribution of the most frequent diagnostic groups in the ATDM among 15- to 39-year-olds according to sex is shown in Figure 3a. The most striking difference between females and males in the 15- to 39-year age range is the much higher frequency of thyroid carcinoma in females (94 females vs. 20 males, *p* < 0.0001) as well as the significantly higher frequency in males for eye and CNS tumors (8 females vs. 26 males, *p* < 0.001), oral cavity and head and neck tumors (2 females vs. 15 males, *p* < 0.001), and Non-Hodgkin lymphoma (8 females vs. 25 males, *p* < 0.01).

The distribution of the most frequent cancers by 5-year age intervals within the 15- to 39-year age range is shown in Figure 3b.

### 3.1. Comparing Cancer Diagnosis in CCCA (ATDM) Patients and the GCR Database

**Figure 4:**
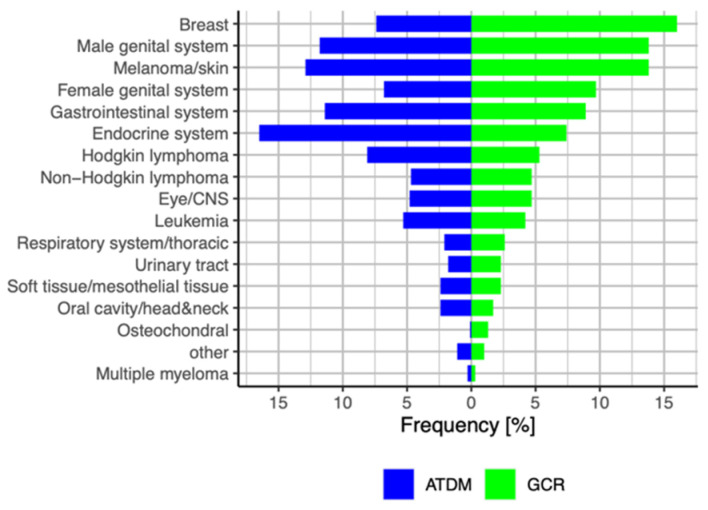
Distribution of cancer types in AYA cancer patients from the Augsburg Tumor Data Management (ATDM) and the Centre for Cancer Registry (GCR) of the Robert-Koch-Institute (RKI), Germany.

Next, we compared our data with the data published in the monograph from the GCR of the RKI in Germany on incidences and outcomes of AYAs aged 15–39 years between 1999 and 2016 [35]. While breast cancer is underrepresented in the Augsburg population and endocrine neoplasia (mostly thyroid cancer) is overrepresented, our data largely reflect the incidences reported by the GCR registry highlighting the unique distribution of the types of cancer that occur in AYAs (shown in Figure 4).

### 3.2. Hereditary Cancer Risk Assessment in AYA Patients

A major challenge beyond treatment of AYAs is the recognition of a CPS. To determine the number of AYAs at risk, we analyzed our data allowing for various clinical criteria.

First, we looked at the family history. Of note, family history assessment was available in only 86 (12.2%) of 704 AYAs, in 44 (51%) of these, family history was rated positive by the treating physician.

Next, we analyzed our data for the presence of multiple primary cancers (MPC). Two or more malignancies were reported in 36 (5.1%) patients. Of those, 27 (75%) patients were diagnosed with their first malignancy prior to the study period and, thus, at a younger age. Median age of those patients was 26 years (range, 2–38 years), 15 patients were male. Six patients were diagnosed before the age of 15. The median time of onset between MPCs was 6 years. Tumors of the male genital system (5 out of 27) and osteochondral tumors (4 out of 27) were most prevalent and 14 out of those 27 patients were treated with chemo- or radiotherapy for their first malignancy.

Nine (25.0%) patients developed a second primary malignancy during the study period.

The occurrence of cancer of older adulthood at unusually early ages is a characteristic of a hereditary cancer predisposition. To this end, we sorted our data into patients with EO-CA and YA-CA analogous to the analysis of Stadler et al. [12] identifying 507 (71.9%) patients with EO-CA.

The occurrence of specific malignancies and certain histopathological subtypes, respectively (shown in Table S1, that have an associated higher CPS rate are an indicator of inherited cancer susceptibility. Looking more closely into the ICD-based diagnosis groups, of 116 patients with cancers of the endocrine system three (2.6%) patients presented with medullary thyroid carcinoma. Of 80 patients with tumors of the gastrointestinal system, 16 (20%) patients were diagnosed with esophageal and gastric cancer, 37 (46%) patients with colorectal cancer, four (5.0%) patients with pancreatic cancer, and one (1.3%) patient with pancreatobiliary carcinoma. According to the study design, all 52 patients with breast cancer were diagnosed below the age of 45 years and, thus, were suspicious of having hereditary breast and ovarian cancer. One patient was diagnosed with SMARC-deficient tumor of the ovary. Of 32 patients with tumors of the central nervous system, 21 (66%) patients were diagnosed with glioma, four (12.5%) patients with medulloblastoma, and one (3.1%) patient each with ependymoma, pineoblastoma, and atypical teratoid/rhabdoid tumor. In addition, 16 patients presented with any type of sarcoma, one patient was diagnosed with a malignant peripheral nerve sheath tumor. Twelve of 13 patients with cancers of the urinary tract were diagnosed with malignancies associated with a higher CPS rate, namely renal cell carcinoma in 10 (77%) patients including four patients with renal cell carcinoma of the papillary and two patients of the clear cell subtype and two (15.4%) patients with papillary ureteric cancer. Noteworthy, of 17 patients with tumors of the oral cavity/head and neck tumors, 10 patients presented with squamous cell carcinoma which may be an indicator of a so far undiagnosed Fanconi anemia [36]. In sum, at least 181 of 704 (25.7%) AYA cancer patients presented with malignancies suspicious of harboring pathogenic germline variants.

## 4. Discussion

The treatment of AYA cancer patients carries unique challenges including a wide spectrum of cancer diagnoses. Our data mirror this wide spectrum comprising about 60 different cancer diagnoses in 704 AYAs. Furthermore, the distribution of cancer types changes significantly from leukemia and lymphoma being the most frequent malignancies at age 15 years to endocrine tumors, gastrointestinal tumors, and melanoma at age 39 years resembling previously published data [24].

Facing recent advances in and the expanded use of massively parallel sequencing and the rapidly increasing literature on CPS, clinician awareness, identification of and referral rates to genetic testing of AYAs, however, are still suboptimal. Several clinical practice guidelines for CPS evaluation have been published to assist physicians in recognizing patients in daily routine [6,7,37]. Evaluation of AYAs, however, has yet not been established in daily practice. To overcome this shortcoming, we retrospectively analyzed data of 704 AYAs referred to our hospital. Only a small number of patients was referred to genetic testing, whereas the vast majority of patients was not evaluated for an underlying CPS.

The family history is considered a cornerstone of CPS evaluation yet. In 87% of patients, however, family history data were not collected at all, which is a common drawback in daily routine. The presence of these data was mainly restricted to patients in whom risk assessment tools for the specific type of cancer were well-established, e.g., breast and colorectal cancer. Of note, recent studies reported inherited PGVs in 48.4% and 55.5% of patients with CPS who would not have been referred to genetic testing based on standard guidelines including family history [9,10].

MPC may be another indicator of CPS. Thirty-six (5.1%) AYAs presented with MPC. As only 6 of these patients were at the age before 15, the majority of AYAs with MPC would have been diagnosed as AYAs already for their first malignancy prior to the study period. This indicates that the occurrence of multiple AYA cancers was considerably more frequent than the previous presence of childhood cancers. Approximately half of the patients with previous childhood cancer were treated with chemo- or radiotherapy suggesting that second malignancy after exposure to previous treatment is certainly a risk factor, although not overly dominant, and needs to be considered when evaluating MPCs in AYAs.

A number of genetic syndromes is associated with an increased susceptibility to MPC particularly at a young age (e.g., Li-Fraumeni syndrome, Lynch syndrome, hereditary breast and ovarian cancer, neurofibromatosis type 1, multiple endocrine neoplasia type 1 and 2, von Hippel-Lindau disease; reviewed in [38]). For example, in germline *TP53* mutation carriers, the risk for a second primary cancer was estimated to be 50% [39], MPC were observed in 43% of patients [40]. The significant association of genetic syndromes with MPC supports referral of all patients with MPCs for genetic counselling.

To immediately compare our data to the data presented by Stadler et al., we applied their definition of EO-CA referring to SEER data [12]. And indeed, in our cohort of AYAs, 72% of patients presented with malignancies typically presenting at later ages, which is in line with EO-CA in 73% of patients reported by Stadler et al. Of those 877 patients with EO-CA reported by Stadler et al., 21% harbored P/LP germline variants in cancer predisposing genes. Of note, high- and moderate-penetrance PGVs were enriched in these patients indicating a high risk of cancer development for at-risk relatives. It deserves mentioning, however, that these categories can be questioned in skewed and exponentially rising age adjusted data and that the definition of EO-CA is heterogenous in the literature. In addition, the definitions of Stadler et al. are useful epidemiologically but not clinically because they are impractical for everyday use. Thus, a biology and histology-based approach may be more useful.

Specific types of cancer such as medullary thyroid cancer and adrenocortical carcinoma have been associated with a higher CPS rate. Current recommendations for genetic counselling and testing, however, are rather conservative and leave demographic changes out of consideration (e.g., deficit of births resulting in small families not fulfilling criteria for genetic testing due to the number of relatives). In addition, genetic testing guidelines are adapted too slowly to recent findings, e.g., germline genetic testing of *BRCA1/2* in patients with pancreatic cancer to facilitate individualized therapies with the PARP inhibitor olaparib [41].

Taking recent publications on germline mutations in certain types of cancer into consideration, patients diagnosed with pancreatic cancer [9,10], cholangiocellular carcinoma [10], urothelial cancer [9,10], renal cell carcinoma [9,10], ovarian and endometrial cancer [10], and esophageal and gastric cancer [10] need to be referred to genetic counselling. In 18.1% to 55% sarcoma patients, germline mutations have been reported [28,42,43,44]. The most frequently affected genes are involved in the DNA damage repair pathway. Thus, all sarcoma patients are in need of at least genetic counselling due to therapeutic considerations. In addition, allowing for the age of AYAs, all patients diagnosed with breast cancer [26,45] and CRC [27,46] need genetic counselling. Most notably, however, of 17 patients with oral cavity/head and neck cancer, 10 patients were diagnosed with squamous cell carcinoma indicating an undiagnosed Fanconi anemia. Considering these recent data on PGVs in specific types of cancer, at least about 26% of AYA cancer patients in our cohort would have been in need of genetic counselling. It should not go unmentioned that the ATDM did not capture detailed data on leukemia and lymphoma diagnosis. Thus, our data were not sufficient for identifying patients diagnosed with leukemia and lymphoma at risk for an underlying CPS.

Noteworthy, the list of types of cancers compiled for this study only demonstrates a snapshot of the current knowledge of hereditary cancer predisposition which will be outdated in the near future. In addition, the list and overview on recent publications certainly is not complete. To keep up with the rapidly evolving and increasingly complex field of genetic susceptibility, systematic approaches for the evaluation of AYA cancer patients by an interdisciplinary team specialized in CPS need to be established at each hospital.

Some CPS tools recently included tumor testing suggestive of an underlying PGV in a cancer predisposing gene as an additional criterion to identify patients in need for genetic germline testing. These criteria include mismatch repair or SDHB-deficient tumors, mutational signatures, and certain somatic mutations with high variant allele frequencies. Data on tumor testing, however, were not recorded in the ATDM and, thus, were not analyzed.

The paramount implication of genetic testing and the knowledge of inherited cancer susceptibility is the great hope for individualized therapies, targeted efforts of tumor surveillance for early detection of subsequent tumors, and cascade testing of at risk relatives. This presupposes clinical actionability of pathogenic variants including their potential as therapeutic targets and utility in cancer prevention. Mandelker et al. reported clinically actionable variants in 182 of 1040 patients (17.5%) with advanced cancer including 149 moderate- to high-penetrance variants [9]. Of those 182 probands with actionable findings, 132 had variants in DNA repair genes, enabling the use of targeted therapy. Samadder et al. reported a lower PGV prevalence of 13.3% among 2984 patients with solid cancers [10]. Of 149 patients with high-penetrance PGVs, 42 (28.2%) had clinically actionable management and treatment changes including targeted therapy (*n* = 21), enrollment in clinical trials (*n* = 2), and surgery (*n* = 18). In the study reported by Idos et al., 12% of 2000 patients harbored PGVs [47]. Sixteen of those tested positive underwent prophylactic surgery. Of note, the studies indicate, that up to half of the inherited variants found by tumor-normal sequencing would not have been detected by traditional well-established approaches to selection for genetic testing. For example, in the study reported by Mandelker et al., 22% of patients with germline *BRCA1/2* variants and 42.8% of patients with mismatch repair gene variants would not have been referred for testing using existing guidelines. This strongly supports a role for genetic testing irrespective of family history in AYAs.

Our analysis strongly displays the urgent need of evaluation for hereditary cancer predisposition in AYA cancer patients and clearly advocates for a more systematic and consistent integration of the screening for hereditary cancer predisposition in AYAs into daily practice. However, our study has several limitations of which only some have been discussed so far. Compared to data of other AYA cancer registry reports, breast cancer and, to a lesser extent, male germ cell tumors were underrepresented and thyroid cancer was overrepresented in our cohort of AYA cancer patients. This variation is most likely due to center effects of this single-center report including on one hand a great number of well-established resident physicians with in-patient beds in the catchment area as well as referral to certified breast cancer centers. On the other hand, the CCCA traditionally has a strong expertise in endocrine surgery attracting patients with thyroid cancer outside the catchment area. This variation may have influenced our results since the proportion of cancers would change with a more typical distribution of AYA cancers. While a higher number of breast cancer patients and a smaller number of patients with thyroid cancer (most of them not fulfilling criteria for genetic counselling) would have increased the number of patients in need of genetic counselling, a higher number of patients with male germ cell tumors would have decreased this number. In addition, the lack of genetic data rendered it impossible to compare clinically identified patients suspicious of an underlying CPS and patients truly harboring pathogenic germline variants in cancer predisposing genes.

## 5. Conclusions

Our data confirm the wide spectrum and specific distribution of cancer diagnosis in AYA cancer patients. It highlights the lacking awareness of hereditary cancer predisposition in AYAs while being at high risk. The knowledge of an underlying inherited cancer susceptibility facilitates individualized therapies and targeted efforts in cancer surveillance and prevention. Evaluation of AYAs for hereditary cancer predisposition needs to be integrated into daily practice.

## Figures and Tables

**Table 1:** Types of cancer associated with CPS used as indicator for need of referral to genetic counselling.

ICD Groups	Specific Types of Cancer
Endocrine system	Thyroid cancer, medullary
	Adrenocortical carcinoma
	Pheochromocytoma, paraganglioma
Melanoma/skin	Basal cell carcinoma, >5
	Basal cell carcinoma, <30 years of age
	Sebaceous neoplasm
Gastrointestinal system	Gastric cancer, diffuse
	Small intestine cancer
	Colon cancer, <50 years of age
	Pancreatobiliary carcinoma
Breast cancer	Breast cancer, unilateral (<36 years of age)
	Breast cancer, bilateral (<51 years of age)
	Breast cancer, male
Female genital system	Ovarian cancer
	Tubal cancer
	Primary peritoneal carcinoma
Leukemia	Acute lymphoblastic leukemia, low hypodiploid
Central nervous system/eye	Choroid plexus carcinoma
	Brain tumor, <46 years of age
	Gangliocytoma, dysplastic cerebellar (Lhermitte-Duclos disease)
	Meningioma, clear-cell
	Schwannoma, ≥2 (non-dermal)
	Endolymphatic sac tumor
	Retinoblastoma
Soft tissue/mesothelial tissue	Rhabdomyosarcoma, embryonal
	Rhabdomyosarcoma, anaplastic
	Soft tissue sarcoma, <46 years of age
	Leiomyoma, cutaneous
Urinary tract	Renal cell carcinoma (<47 years of age)
	Renal cell carcinoma, papillary
	Renal cell carcinoma, clear cell tubulo-papillary
	Collecting-duct carcinoma
	Ureteric cancer
Osteochondral	Osteosarcoma, <46 years of age
Other	Rhabdoid tumor
	Hemangioblastoma
	Pineoblastoma

## Data Availability

No new data were created or analzyed in this study. Data sharing is not applicable to this article.

## Supplementary Materials

The following are available online at https://www.mdpi.com/article/10.3390/cancers13123033/s1, Table S1: Abstract of recent data on cancer susceptibility and the proportion of patients with inherited pathogenic germline variants depending on cancer type.
