# Supplementary material for: A Retrospective 5-Year Single Center Study Highlighting the Risk of Cancer Predisposition in Adolescents and Young Adults
Source: Cancers (Basel). 2021 Jun 17;13(12):3033. doi: 10.3390/cancers13123033 (PMC8234548; doi:10.3390/cancers13123033)
Supplement: Supplementary file 1 [file cancers-13-03033-s001.zip › cancers-1245006-supplementary.pdf]

Table S1: Abstract of recent data on cancer susceptibility and the proportion of patients with inherited pathogenic germline variants depending on cancer type.

	Tumor entities and associated relevant CPS and CPG	*Proportion of patients with inherited pathogenic variants*
All cases		– 19.7% of 1040 pts[1]<br>– 21% of 877 early-onset cancer pts *(BRCA1/2, CHEK2, ATM)*[2]<br>– 13% of 324 young-adult cancer pts *(TP53, SDHA)*[2]
Endocrine system	***Medullary thyroid carcinoma***<br>– Multiple Endocrine Neoplasia Type 2 *(RET)*<br>***Papillary thyroid carcinoma***<br>– DICER1 syndrome *(DICER1)*	***Familial non-medullary TC***<br>– 3–9%[3]
Skin	***Melanoma***<br>– Familial melanoma *(CDKN2A, CDK4, MC1R, BAP1, POT1, TERF2IP, ACD, TERT)*	***Melanoma***<br>1 of 3 pts[1]
Male genital system		***Prostate cancer***<br>– 19.6% of 362 pts *(BRCA1/2, ATM, CHEK2, PMS2, FLCN, PALB2)*[1]<br>***Testicular germ cell tumor***<br>– 22 of 205 pts (DNA repair genes, 1/one-third in *CHEK2)*[4]
Gastrointestinal system	***Colorectal cancer***<br>– Hereditary non-polyposis colon cancer (MMR genes)<br>– Cowden´s syndrome/PTEN hamartoma tumor syndrome *(PTEN)*<br>– Juvenile polyposis syndrome *(SMAD4, BMPR1A, ENG)*<br>– Peutz–Jeghers syndrome *(STK11)*<br>– Bannayan–Riley–Ruvalcaba Syndrome *(unknown)*<br>– (Familial) Adenomatous polyposis syndromes *(APC)*<br>– Gardner´s syndrome *(APC)*<br>– Turcot´s syndrome *(APC, MLH1, PMS2)*<br>– Muir–Torre's syndrome *(MLH1, MSH2)*<br>– Oldfield´s syndrome *(APC)*<br>***Hepatocellular carcinoma***<br>– Hereditary tyrosinemia *(FAH, TAT, HPD)*<br>– Glycogen storage disease *(G6PC1, SLC37A4)*	***Biliary tract cancer***<br>– 16.0% of 131 pts *(BRCA1/2, PALB2, BAP1, PMS2. ATM, MITF, NBN)*[5]<br>– 22.2% of 27 pts *(BRCA2)*[1]<br>***Pancreatic cancer***<br>– 16.0% of 131 pts *(BRCA1/2, ATM, PALB2, MLH1, MSH2, MSH6, PMS2, CDKN2A, TP53)*[6]<br>– 25.0% of 176 pts *(BRCA1/2, CHEK2, ATM)*[1]<br>***Colon cancer***<br>– 9.2% of 65 pts[1]<br>***Small-bowel***<br>– 2 of 5 pts[1]<br>***Esophagogastric carcinoma***<br>– 17.6% of 34 pts *(BRCA2, ATM)*[1]

	– Alpha 1-antitrypsin deficiency *(SERPINA1)*	
Lymphoma	– Immunodeficiencies<br>– DNA repair defects<br>– Familial lymphoma cases	
Breast	– Hereditary breast and ovarian cancer *(BRCA1, BRCA2)*<br>– Li–Fraumeni syndrome *(TP53)*<br>– Muir–Torre’s syndrome *(MLH1, MSH2)*<br>– Cowden´s syndrome *(PTEN, KLLN, SDHB, SDHC, SDHD, AKT1, PIK3CA)*	– 16.8% of 101 pts[1]
Female genital system	***Uterine carcinoma***<br>– DICER1 syndrome *(DICER1)*<br>***Ovarian Sertoli–Leydig cell tumor***<br>– DICER1 syndrome *(DICER1)*	***Ovarian cancer***<br>– 31.6% of 19 pts *(PALB2)*[1]<br>***Endometrial cancer***<br>– 16% of 25 pts[1]
Leukemia	***Acute lymphoblastic leukemia***<br>– Trisomy 21 (n.a.)<br>– Neurofibromatosis type 1 *(NF1)*<br>– Bloom syndrome *(BLM)*<br>– Shwachman Diamond syndrome *(SBDS)*<br>– Ataxia telangiectasia *(ATM)*<br>– PAX5 syndrome *(PAX5)*<br>– Li-Fraumeni syndrome *(TP53)*<br>***Acute myeloid leukemia/MDS***<br>– Trisomy 21 (n.a.)<br>– Fanconi anemia *(FANCA, FANCB, FANCC, FANCD1, FANCD2, FANCE, FANCF, FANCG, FANCI, FANJ, FANCL, FANCM, FANCN, FANCO)*<br>– Neurofibromatosis type 1 *(NF1)*<br>– Bloom syndrome *(BLM)*<br>– Shwachman Diamond syndrome *(SBDS)*<br>– Familial monosomy 7 (n.a.)<br>– Severe congenital neutropenia (Kostman syndrome) *(ELANE, GFI1, HAX1, G6PC3, VPS45, WASP)*<br>– Familial MDS/AML *(DDX41)*	

	– Lifelong thrombocytopenia *(RUNX1, ANKRD26, ETV6)*<br>– MIRAGE syndrome *(SAMD9/L)*<br>– GATA2 deficiency syndrome *(GATA2)*<br>– Inherited BMF syndromes (various genes)	
Central nervous system	***Glioma***<br>– Neurofibromatosis type 1 and 2 *(NF1, NF2)*<br>– Tuberous sclerosis type 1 and 2 *(TSC1, TSC2)*<br>– Von Hippel–Lindau syndrome *(VHL)*<br>– Li-Fraumeni syndrome *(TP53)*<br>– Turcot's syndrome *(APC, MLH1, PMS2)*<br>***Medulloblastoma***<br>– Li-Fraumeni syndrome (*TP53*)<br>– Gorlin's syndrome *(PTCH1, SUFU)*<br>– Turcot's syndrome *(APC, MLH1, PMS2)*<br>***Meningioma***<br>– Neurofibromatosis type 1 and 2 *(NF1, NF2)*<br>– Gorlin's syndrome *(PTCH1, SUFU)*<br>***Acoustic neuroma***<br>– Neurofibromatosis type 2 *(NF2)*<br>***Schwannoma***<br>– Neurofibromatosis type 2 *(NF2)*<br>***Ependymoma***<br>– Neurofibromatosis type 2 *(NF2)*<br>– Tuberous sclerosis type 1 and 2 *(TSC1, TSC2)*<br>– Multiple endocrine neoplasia type 1 *(MEN1)*<br>***Subependymal giant cell astrocytoma***<br>– Tuberous sclerosis type 1 and 2 *(TSC1, TSC2)*<br>***Hamartoma***<br>– Tuberous sclerosis type 1 and 2 *(TSC1, TSC2)*<br>***Cerebellar hemangioblastoma***<br>– Von Hippel–Lindau syndrome *(VHL)*<br>***Atypical teratoid/rhabdoid tumor***	***Medulloblastoma***<br>– 6% of 1,022 pts *(APC, BRCA2, PALB2, PTCH1, SUFU, TP53)*[8]

	– Rhabdoid tumor predisposition syndrome type 1 and 2 *(SMARCB1, SMARCA4)*<br>***Pineoblastoma***<br>– Hereditary retinoblastoma *(RB1)*<br>– DICER1 syndrome *(DICER1)*<br>***Pituitary adenoma***<br>– Multiple endocrine neoplasia type 1 *(MEN1)*	
Respiratory system/ thoracic		***Non-small cell lung cancer***<br>– 1 of 2 pts[1]
Urinary tract		***Renal cancer***<br>– 16.4% of 140 pts[1]<br>***Bladder cancer*** *(including urothelial carcinoma)*<br>– 56.3% of 16 pts[1]
Bone/ soft tissue/ mesothelial tissue	***Osteosarcoma***<br>– Paget disease *(SQSTM1)*<br>– Hereditary retinoblastoma *(RB1)*<br>– Rothmund–Thomson syndrome *(RECQL4)*<br>– Werner syndrome *(WRN)*<br>– Bloom syndrome *(BLM)*<br>– Li-Fraumeni syndrome *(TP53)*<br>***Chondrosarcoma***<br>– Marfucci's syndrome *(somatic mosaicism in IDH1, IDH2, PTHR1)*<br>– Ollier's disease *(somatic mosaicism in IDH1, IDH2, PTHR1)*<br>– Hereditary multiple osteochondromatosis/exostosis *(EXT1, EXT2)*	***Sarcoma***<br>– 18.1% of 1,201 pts[2]<br>– 55% of 1,162 pts *(TP53, ATM, BRCA2, ATR)*[9]<br>***Osteosarcoma***<br>– 28.0% of 1004 pts *(TP53, CDKN2A, MEN1, VHL, POT1, APC, MSH2, ATRX)*[10]<br>***Sporadic sarcoma***<br>– 13.6% of 66 pts *(ATM, BRCA2, ERCC4, FANCC, FANCE, FANCI, MSH6, POLE, SDHA, TP53)*[11]

Legend: ACC, adrenocortical carcinoma; CPS, cancer predisposition syndrome; CPG, cancer predisposition gene; GIST, gastrointestinal stromal tumor; PPGL, pheochromocytoma paraganglioma; n.a., not applicable; pts, patients

References

1. Mandelker D, Zhang L, Kemel Y, et al. Mutation Detection in Patients With Advanced Cancer by Universal Sequencing of Cancer-Related Genes in Tumor and Normal DNA vs Guideline-Based Germline Testing. *JAMA : the journal of the American Medical Association.* 2017;318(9):825-835.

2. Stadler ZK, Maio A, Padunan A, et al. Abstract 1122: Germline mutation prevalence in young adults with cancer. *Cancer research.* 2020;80(16 Supplement):1122-1122.
3. Klubo-Gwiezdzinska J, Yang L, Merkel R, et al. Results of Screening in Familial Non-Medullary Thyroid Cancer. *Thyroid.* 2017;27(8):1017-1024.
4. AlDubayan SH, Pyle LC, Gamulin M, et al. Association of Inherited Pathogenic Variants in Checkpoint Kinase 2 (CHEK2) With Susceptibility to Testicular Germ Cell Tumors. *JAMA Oncol.* 2019;5(4):514-522.
5. Maynard H, Stadler ZK, Berger MF, et al. Germline alterations in patients with biliary tract cancers: A spectrum of significant and previously underappreciated findings. *Cancer.* 2020;126(9):1995-2002.
6. Rainone M, Singh I, Salo-Mullen EE, Stadler ZK, O'Reilly EM. An Emerging Paradigm for Germline Testing in Pancreatic Ductal Adenocarcinoma and Immediate Implications for Clinical Practice: A Review. *JAMA Oncol.* 2020;6(5):764-771.
7. Duan L, Grunebaum E. Hematological Malignancies Associated With Primary Immunodeficiency Disorders. *Clinical immunology.* 2018;194:46-59.
8. Waszak SM, Northcott PA, Buchhalter I, et al. Spectrum and prevalence of genetic predisposition in medulloblastoma: a retrospective genetic study and prospective validation in a clinical trial cohort. *The Lancet Oncology.* 2018;19(6):785-798.
9. Ballinger ML, Goode DL, Ray-Coquard I, et al. Monogenic and polygenic determinants of sarcoma risk: an international genetic study. *The Lancet Oncology.* 2016;17(9):1261-1271.
10. Mirabello L, Zhu B, Koster R, et al. Frequency of Pathogenic Germline Variants in Cancer-Susceptibility Genes in Patients With Osteosarcoma. *JAMA Oncol.* 2020.
11. Chan SH, Lim WK, Ishak NDB, et al. Germline Mutations in Cancer Predisposition Genes are Frequent in Sporadic Sarcomas. *Sci Rep.* 2017;7(1):10660.
